# Critical factors for the bulk adhesion of engineered elastomeric proteins

**DOI:** 10.1098/rsos.171225

**Published:** 2018-05-09

**Authors:** M. Jane Brennan, Sydney E. Hollingshead, Jonathan J. Wilker, Julie C. Liu

**Affiliations:** 1 Davidson School of Chemical Engineering, Purdue University, West Lafayette, IN 47907, USA; 2 Department of Chemistry, Purdue University, West Lafayette, IN 47907, USA; 3 School of Materials Engineering, Purdue University, West Lafayette, IN 47907, USA; 4 Weldon School of Biomedical Engineering, Purdue University, West Lafayette, IN 47907, USA

**Keywords:** biomimetic, adhesive, lap shear, elastin, recombinant protein

## Abstract

Many protein-based materials, such as soy and mussel adhesive proteins, have been the subject of scientific and commercial interest. Recently, a variety of protein adhesives have been isolated from diverse sources such as insects, frogs and squid ring teeth. Many of these adhesives have similar amino acid compositions to elastomeric proteins such as elastin. Although elastin is widely investigated for a structural biomaterial, little work has been done to assess its adhesive potential. In this study, recombinant elastin-like polypeptides were created to probe the factors affecting adhesion strength. Lap shear adhesion was used to examine the effects of both extrinsic factors (pH, concentration, cross-linker, humidity, cure time and cure temperature) and intrinsic factors (protein sequence, structure and molecular weight). Of the extrinsic factors tested, only humidity, cure time and cure temperature had a significant effect on adhesion strength. As water content was reduced, adhesion strength increased. Of the intrinsic factors tested, amino acid sequence did not significantly affect adhesion strength, but less protein structure and higher molecular weights increased adhesion strength directly. The strengths of proteins in this study (greater than 2 MPa) were comparable to or higher than those of two commercially available protein-based adhesives, hide glue and a fibrin sealant. These results may provide general rules for the design of adhesives from elastomeric proteins.

## 1. Introduction

Protein-based adhesion has been the subject of recent and historical scientific interest. Soy protein has been used commercially for a renewable, low-cost wood glue for nearly a century since the original patent in 1923 [1,2]. Similar adhesives can be created from other crops, including sorghum, camelina and canola [3]. For these glues, adhesive performance derives primarily from mechanical interlocking between protein chains and the porous wood structure with contributions from hydrogen bonding and van der Waals forces [2]. Thus, adhesion strength is directly related to a variety of factors, including protein denaturation, glue viscosity, particle size and substrate physical properties [2,4].

Mussel adhesive proteins (MAPs) have also received significant interest from the scientific community for their ability to form adhesive bonds in wet environments [5,6]. The wet adhesion strength of MAPs is largely due to the non-canonical amino acid 3,4-dihydroxyphenylalanine (DOPA) [7–9]. DOPA provides bulk adhesive strength through the combination of adhesive interactions with the substrate and cohesive interactions from cross-linking [8,9]. The presence of many charged lysine residues in MAPs has also been cited for potential contributions to MAP adhesion strength [10–13].

More recently, other natural protein-based adhesives have been isolated and characterized. For example, the frog *Notaden bennetti* secretes a sticky protein solution for a defence mechanism [14], and the velvet worm captures prey with a similar protein solution [15]. A wide variety of insects also produce protein-based glues. Gum moths, blowflies and ladybirds use protein glues for egg attachment, and spittle bugs, froghoppers and lerps produce protein-based materials for protection [16]. If raised above their glass transition temperature, the structural proteins from squid ring teeth formed a strong underwater adhesive [17]. Interestingly, the reported shear adhesion strengths (1–2 MPa) for these protein adhesives were all quite similar [14,16,17]. Furthermore, many of these protein adhesives have similar amino acid compositions: glycine is nearly always over-represented, and proline and serine are often present at unusually high mole percentages [14–17].

The amino acid compositions of these natural adhesive proteins are also similar to that of elastin, which is normally enriched in both glycine and proline [18]. Elastin is an elastomeric protein renowned for its structural properties of low stiffness, high extensibility and high resilience [18–21]. In addition, elastin can be produced recombinantly with high yields in *Escherichia coli* [19]. Recombinant design provides for precise control over protein molecular weight and amino acid sequence. This design flexibility allows for investigation into the effects of small protein sequence changes in protein function [22–24]. Despite the fact that elastomeric proteins have been widely studied for biomaterials, only a few studies have examined the adhesive properties of elastomeric proteins [25–27].

Because of their similarities to natural protein-based adhesives, we hypothesized that elastin-like polypeptides (ELPs) would have significant bulk adhesion strength. Furthermore, we wanted to assess the potential of ELPs for adhesive materials and understand the important factors contributing to the adhesive qualities of these and other natural protein-based glues. In this study, a set of recombinant ELPs with varied guest residue compositions was systematically investigated to observe the effects of various extrinsic and intrinsic factors on the lap shear strength of protein adhesives.

## 2. Material and methods

### 2.1. Reagents

All chemicals were purchased from Sigma-Aldrich (St. Louis, MO) or Avantor Performance Materials (Center Valley, PA) unless stated otherwise. Water was ultra-purified with a Milli-Q ultrapurification system (Millipore, Billerica, MA).

### 2.2. Protein design and cloning

**Figure 1.**
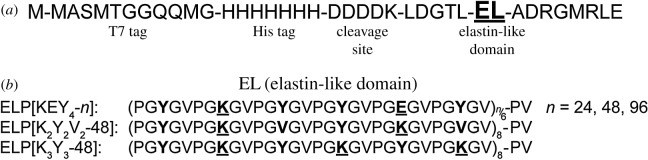
Complete amino acid sequences of the ELPs used in this study. (*a*) At their N-terminus, all proteins contained a T7 tag for western blot identification, a 7×His tag for nickel column purification and an enterokinase cleavage site. (*b*) Within the elastomeric domains, ELP guest residues are in bold, and charged residues are underlined.

DNA sequences were designed using the Geneious software (Biomatters, Inc., San Francisco, CA). Complete amino acid sequences for the final protein constructs are shown in figure 1. Predicted protein isoelectric points (pI) were estimated with the Geneious software. The grand average of hydropathicity (GRAVY), based on the scale by Kyte & Doolitte [28], was calculated for each protein using the ExPASy ProtParam tool (http://web.expasy.org/protparam).

The elastin-based proteins ELP[KEY_4_-24], ELP[KEY_4_-48], ELP[KEY_4_-96] and ELP[K_3_Y_3_-48] were constructed with a cloning scheme modified from one previously developed by our laboratory [29]. The new scheme used AgeI and AvaI restriction enzymes (New England Biolabs, Ipswich, MA) to achieve seamless repeats of the elastin-like sequence. Standard molecular cloning techniques were used throughout [30]. The protein ELP[K_2_Y_2_V_2_-48] was developed previously and was referred to as ELY_16_ [27].

### 2.3. Protein expression and purification

Each of the proteins was cloned and then overexpressed using the T7 expression system. ELP[KEY_4_-48] and ELP[K_3_Y_3_-48] were purified using a temperature cycling protocol similar to others used to purify ELPs [31]. The remaining ELPs were purified using nickel affinity chromatography. Purity was assessed using densitometry analysis and matrix-assisted laser desorption/ionization time-of-flight (MALDI-TOF) mass spectrometry. Complete details for expression and purification are available in the electronic supplementary material.

### 2.4. Lap shear adhesion

Bulk lap shear adhesion bonding on aluminium was performed following a modified version of the ASTM D1002 standard, as previously described [32,33]. Briefly, aluminium substrates were prepared using ASTM standard D2651-01 for cleaning [34]. Protein was resuspended at 150 mg ml^−1^ in water (unless otherwise specified), and 5 µl of this solution was spread onto each aluminium substrate using a pipette or spatula. Tris(hydroxymethyl) phosphine (THP; Strem Chemicals, Newburyport, MA) was used to cross-link primary amine groups. For all cross-linked protein samples, protein was resuspended at 167 mg ml^−1^, and cross-linker solution was added to make a final protein concentration of 150 mg ml^−1^. The concentration of cross-linker solution was calculated based on the ratio of hydroxyl groups to the number of primary amines in the protein. Titebond Liquid Hide Wood Glue was tested by applying an equivalent weight of glue solids (1.5 mg per test) based on a 41.3 wt% water content (previously determined by C. L. Jenkins 2015, personal communication). Tisseel (donated by Baxter Biosurgery, Deerfield, IL) was prepared according to the manufacturer's directions and tested by applying an equivalent protein content (1.5 mg per test).

Substrates were overlapped with an area of 1.2 × 1.2 cm and were cured for 6 h at 37°C (unless otherwise specified). Humid curing conditions were created by covering the substrates with a layer of damp paper towels followed by a layer of plastic wrap to prevent drying. Lap shear bond strengths were measured using an Instron 5544 Materials Testing System (Norwood, MA) with a loading rate of 2 mm min^−1^ and a 2000 N load cell. Maximum force was divided by overlap area to determine the adhesion strength. When investigating the effects of pH, concentration, cross-linker, moisture, cure time and cure temperature, five samples were tested for each condition. For all other conditions, 10 samples were tested.

Mechanical properties were estimated from the lap shear data examining the effect of moisture on adhesion. Young's modulus was calculated as the slope of the stress–strain curve in the linear region prior to breaking. Toughness was calculated as the area under the entire stress–strain curve.

### 2.5. Circular dichroism

The secondary structure of proteins in solution (0.1–0.2 mg ml^−1^ in water or 3 M urea) was determined using a Jasco-815 circular dichroism (CD) spectrometer (Halifax, Nova Scotia, Canada) with the following parameters: 1 mm path length, 1 nm data pitch, 2 nm bandwidth and 100 nm min^−1^ scanning speed. Each spectrum, including the baseline spectra of water and 3 M urea, was averaged from five scans.

### 2.6. Thermogravimetric analysis

The residual water content during curing at 37°C was determined via thermogravimetric analysis (TGA). For each cure time tested, 20 µl of ELP[KEY_4_-48] at 150 mg ml^−1^ in water was pipetted into a 6.7 × 2.7 mm aluminium pan (Thermal Support, Hayesville, NC). The sample was then heated in a TGA Q50 (TA Instruments, New Castle, DE) to 37°C at a rate of 5°C min^−1^ and held at 37°C for the duration of the cure. Finally, the sample underwent a temperature ramp to 200°C at a rate of 20°C min^−1^. Water content was calculated from the weight loss that occurred near 100°C. Throughout the experiment, the sample was purged with nitrogen gas at a rate of 40 ml min^−1^.

### 2.7. Statistical analysis

Adhesion data are represented with the mean *±* standard deviation. First, outliers were removed from the data after assessment with Grubbs' test. Next, equality of variance was evaluated with Levene's test. Statistically significant differences were determined by one-way ANOVA followed by Tukey's honest significant difference (HSD) or the Games–Howell (for unequal variances) post hoc test. The normality of ANOVA residuals was assessed with the Komogorov–Smirnov test. In the case of non-normally distributed residuals, the original data were transformed according to the Box–Cox or Johnson method before repeating the above analysis. Statistical difference between two groups was determined with an unpaired *t*-test. Statistical analyses were performed with Minitab 17 (State College, PA) or GraphPad online software (La Jolla, CA). A value of *p ≤ *0.05 was considered significant.

## 3. Results

### 3.1. Protein design, expression and purification

In this study, we designed and produced a system of ELPs to probe the effects of protein design on adhesion (see figure 1 and table 1 for details). Three ELPs were designed with varying charged residue content. Hydrophobic tyrosine and valine residues were used to maintain a similar average hydrophobicity based on the scale developed by Urry & Parker [35] and Urry [36]. The ELPs were named following the method previously described by Chilkoti's laboratory [37,38]. Briefly, each ELP was designated as ELP[A*_i_*B*_j_*C*_k _*− *n*], in which A, B and C refer to guest residues (X) of the VPGXG pentapeptide. Subscripts *i*, *j* and *k* describe the numbers of each guest residue used within a group of six pentapeptides, and *n* refers to the total number of pentapeptides in the protein. For example, ELP[KEY_4_-96] contained 96 total VPGXG pentapeptides in which 1/6 of the guest residues were K, 1/6 were E and 4/6 were Y. One of the ELPs, ELP[KEY_4_-*n*], was produced with 24, 48 and 96 total pentapeptides to probe the effect of protein molecular weight.

**Table 1.** Detailed information on recombinant proteins examined in this study.

protein	predicted pI	molecular weight (kDa)	hydropathicity (GRAVY)	expression strain	yield (mg l^−1^)^a^
ELP[KEY_4_-*n*]					
*n* = 24	6.38	15.5	−0.321	BL21(DE3)pLysS	62
*n* = 48	6.39	26.6	−0.209	Rosetta2(DE3)pLysS	358
*n* = 96	6.40	48.8	−0.140	BL21(DE3)	47
ELP[K_2_Y_2_V_2_-48]	10.11	25.5	0.095	BL21-CodonPlus-(DE3)-RIPL	71
ELP[K_3_Y_3_-48]	10.23	26.3	−0.295	BL21(DE3)pLysS	17

^a^Yield was calculated per litre of bacterial culture.

Final purified yields of the proteins are shown in table 1. Expression and purification were confirmed by sodium dodecyl sulfate–polyacrylamide gel electrophoresis and western blot (electronic supplementary material, figure S1). Protein molecular weight was confirmed by MALDI-TOF (electronic supplementary material, figure S2). Protein composition was confirmed by amino acid analysis (electronic supplementary material, tables S1–S5).

### 3.2. Effect of extrinsic factors on bulk adhesion

To examine the effect of pH, concentration, cross-linker, humidity, cure time and cure temperature, lap shear adhesion testing was performed on a single protein, ELP[KEY_4_-48]. Out of the variety of proteins used in this study, this particular protein was chosen as a baseline for extrinsic factor testing due to its high expression yield and intermediate hydropathicity (table 1).

**Figure 2.**
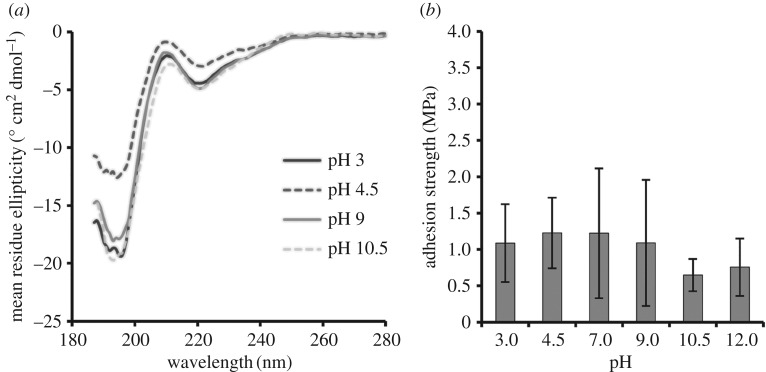
Effect of pH on (*a*) secondary structure and (*b*) bulk adhesion strength of ELP[KEY_4_-48]. (*a*) CD spectrometry was performed on protein solutions at pH 3, 4.5, 9 and 10.5. Secondary structure did not vary significantly with pH. Negative peaks at 198 nm and 220 nm indicate unstructured and β-II turn secondary structure, respectively. (*b*) Bulk adhesion testing between aluminium substrates was performed with protein at pH 3, 4.5, 7, 9, 10.5 and 12. Adhesion strengths did not demonstrate significant variation with pH when assessed by one-way ANOVA followed by Tukey's HSD post hoc analysis.

The pH can affect protein charge, solubility and secondary structure and thereby affect adhesive and cohesive interactions. The overall charge as a function of pH was estimated for ELP[KEY_4_-48] (electronic supplementary material, figure S3), and the secondary structure was examined with CD (figure 2*a*). At a pH below 4 or above 10, the protein was highly charged, was soluble in aqueous solution and, although largely unstructured (negative peak at 198 nm), did exhibit β-II turn structure (negative peak at 220 nm) characteristic of ELPs [39]. At intermediate pH values (approx. 4.5 and approx. 9), ELP[KEY_4_-48] again displayed β-II turn structure and was moderately charged. At a relatively neutral pH, the protein was near its isoelectric point and was completely insoluble and aggregated in aqueous solution. Therefore, we were unable to obtain spectra near neutral pH. Spectra could also not be recorded at pH 12 due to the interference of the high concentrations of base required.

The bulk adhesive strength of ELP[KEY_4_-48] was tested at pH 3, 4.5, 7, 9, 10.5 and 12 (figure 2*b*). Average strengths ranged from 0.65 MPa at pH 10.5 to 1.2 MPa at pH 4.5 and 7. Despite the strong effect that pH had on protein charge, solubility and structure, there were no statistically significant differences in adhesion strengths across the range of pH values tested.

**Figure 3.**
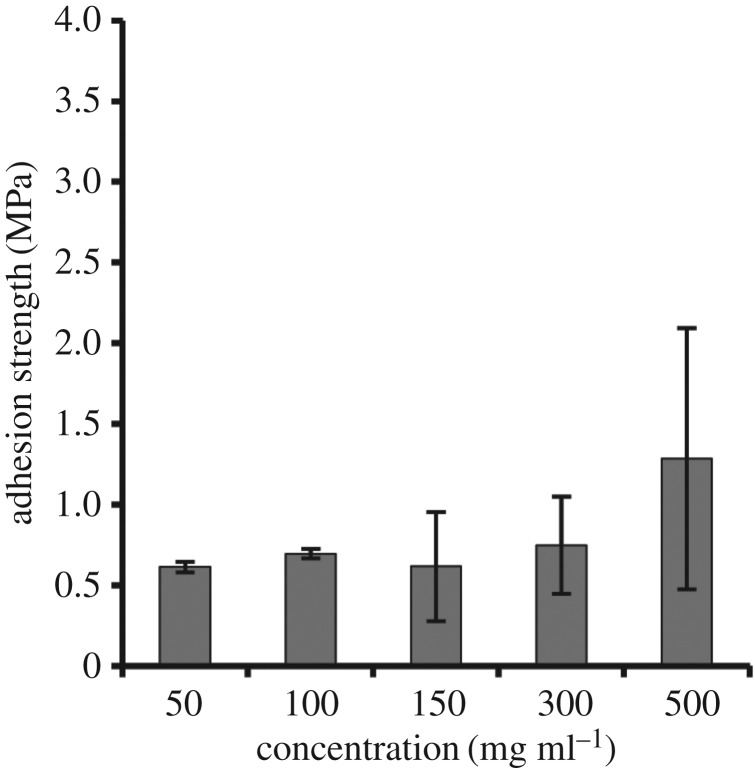
Effect of ELP[KEY_4_-48] protein concentration on bulk adhesion strength between aluminium substrates. Varying the concentration of protein from 50 to 500 mg ml^−1^ resulted in no change to strength when assessed by one-way ANOVA followed by Tukey's HSD post hoc analysis.

The next extrinsic factor tested was protein concentration (figure 3). This property can affect the solution viscosity and bond thickness and, therefore, could have an effect on adhesion strength [40]. Although the concentration of ELP[KEY_4_-48] was varied from 50 to 500 mg ml^−1^, adhesion strengths were not significantly different from each other. It should be noted that it is possible that adhesion strength might increase with very high concentrations of protein, but higher concentrations could not be tested due to a lack of protein solubility.

**Figure 4.**
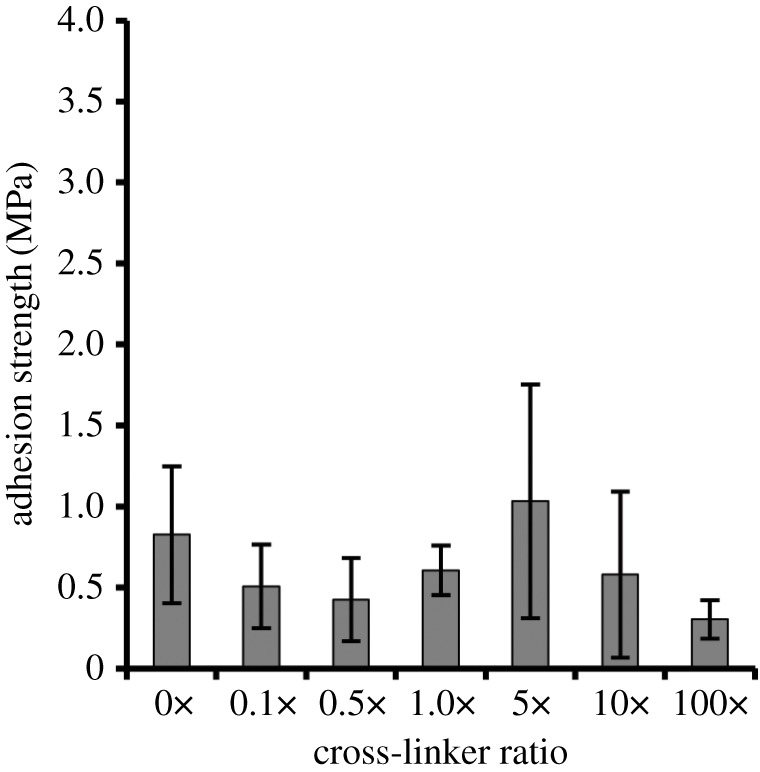
Effect of THP cross-linker on the bulk adhesion strength of ELP[KEY_4_-48]. The cross-linker stoichiometry (THP reactive hydroxyl groups to protein primary amines) was varied between 0× and 100×, but no significant changes in the adhesion strength were detected when assessed by one-way ANOVA followed by Tukey's HSD post hoc analysis.

Bulk adhesion is a balance of adhesive and cohesive interactions between the glue and the substrate [41]. The addition of a cross-linking agent could change this balance and thus affect the adhesion strength. The cross-linker used in this study, THP, reacts with primary amine groups on the protein via condensation reactions in relatively mild conditions with water as a by-product. When testing the effects of cross-linker on adhesion strength, the cross-linker stoichiometry of reactive THP hydroxyl groups to primary amines on ELP[KEY_4_-48] was varied between 0× and 100× (figure 4). There were no significant differences in bulk adhesion strengths across the cross-linker ratios tested.

**Figure 5.**
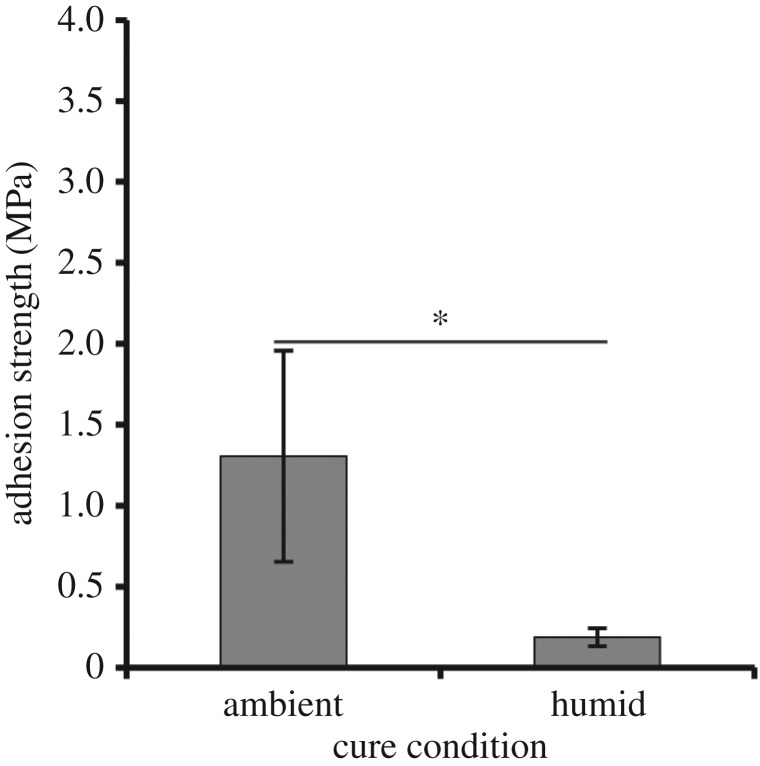
Effect of a humid cure environment on bulk adhesion strength. ELP[KEY_4_-48] was cured at 37°C in both ambient and highly humid environments. Humid curing decreased bulk adhesion strength of the protein significantly. The ambient cure value is the same as that shown in figure 2*b* at pH 3. A statistically significant difference (unpaired *t*-test, *p < *0.05) is indicated by the asterisk.

Historically, bonding in the presence of moisture has been one of the key challenges for adhesive development. To determine the effect moisture might have on the performance of elastomeric proteins, ELP[KEY_4_-48] was cured in a highly humid environment (figure 5). As might be expected, samples cured in humid conditions demonstrated significantly reduced adhesion strength (0.19 MPa) compared with an analogous test under ambient conditions (1.31 MPa). We observed that samples cured in a humid environment retained visible moisture, whereas those cured in ambient air had visibly dried out upon testing. Given the significant differences in adhesion strength due to moisture, we have also included representative stress–strain curves (electronic supplementary material, figure S4) and calculated the Young's moduli and toughness values (electronic supplementary material, table S6) to show the effect of moisture on material properties during adhesion testing.

**Figure 6.**
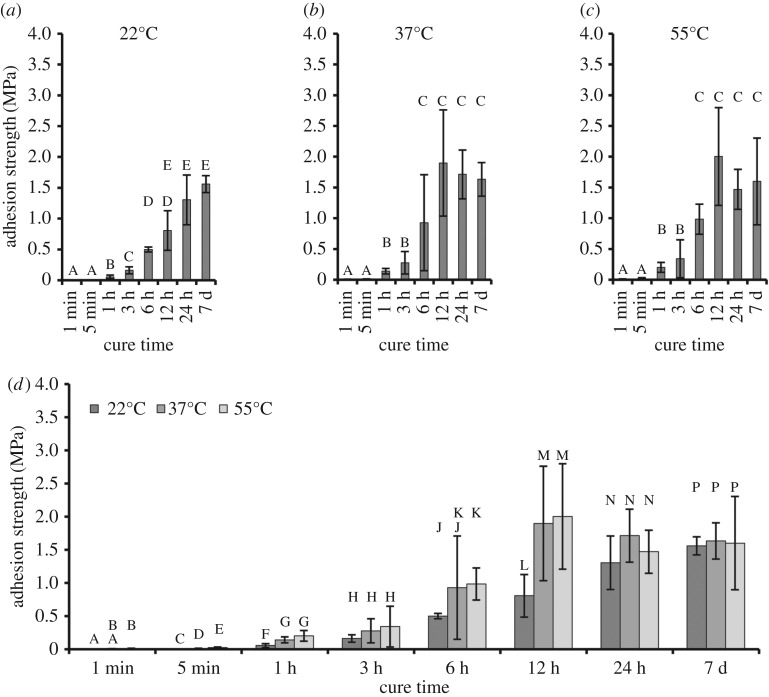
Effect of cure time and temperature on the bulk adhesion of ELP[KEY_4_-48]. Adhesion strengths versus cure times are shown at (*a*) 22°C, (*b*) 37°C and (*c*) 55°C. Groups with identical letters are statistically similar (*p* *>* 0.05) when determined by one-way ANOVA followed by Tukey's HSD post hoc analysis. (*d*) The same data are grouped by cure time. Groups that share a letter are statistically similar (*p* *>* 0.05) when determined by a one-way ANOVA (performed within a single time point) followed by either Tukey's HSD or the Games–Howell post hoc test. Adhesion strength increased with cure time and temperature up to 6–12 h, after which adhesion strength remained constant at all temperatures. Two-way ANOVA results indicate that the relative adhesion strengths varied according to 22°C *<* 37°C, 55°C and 1 min, 5 min *<* 1 h *<* 3 h *<* 6 h *<* 12 h, 24 h, 7 days.

Other curing factors such as time and temperature can also be critical to final adhesion strengths. In this study, samples were cured in ambient conditions for eight different times (1 min, 5 min, 1 h, 3 h, 6 h, 12 h, 24 h and 7 days) at each of three different temperatures (22°C, 37°C and 55°C). Results are shown in figure 6. When a two-way ANOVA was used, both cure time and temperature were significant factors for adhesion strength. With regard to temperature, 22°C resulted in significantly lower strengths than either 37°C or 55°C. With regard to time, 1 and 5 min cures were statistically equivalent. The 12 h, 24 h and 7 days periods were also statistically equivalent. Samples cured for 1, 3 and 6 h were significantly different from each other and also from samples cured for very short (less than 1 h) or very long (greater than 6 h) times. One-way ANOVA allowed closer examination of the trends identified by two-way ANOVA. Similar to what was identified in two-way ANOVA, higher temperatures increased adhesion strengths, but this effect was only found at short cure times (less than or equal to 6 h). At long cure times (greater than or equal to 12 h), this phenomenon was no longer observed.

The increase in adhesion strength with cure time was most likely related to the sample water content. To test this hypothesis, TGA was performed on a protein solution at the same concentration and pH (table 2). This solution was cured at 37°C for 2, 5, 30, 60 and 100 min. When the cure time at 37°C was increased from 2 to 100 min, the water content remaining in the sample decreased from 75.3% to 1.3%. The TGA results showed that significant water loss occurred over time at 37°C. However, the time frame for water loss did not match that seen in lap shear adhesion. This discrepancy was most likely due to the differences in exposed surface area of the open pan during TGA data collection versus the solution residing between two overlapped aluminium substrates for adhesion tests. Combined with the adhesion results from a humid cure, the TGA results demonstrated that water loss was likely to be responsible for the increase in adhesion strength with cure time and temperature.

**Table 2.** TGA results.

cure time (min)	remaining water content (%)
2	75.3
5	71.4
30	19.5
60	2.8
100	1.3

### 3.3. Effect of intrinsic factors on bulk adhesion

To examine the influences of amino acid composition, structure and molecular weight, lap shear adhesion testing was performed using different recombinant proteins. Unless stated otherwise, all intrinsic factors were tested with 10 replicates of 150 mg ml^−1^ of protein cured at 37°C for 24 h. These cure conditions were based on the optimum found from the extrinsic factor tests.

**Figure 7.**
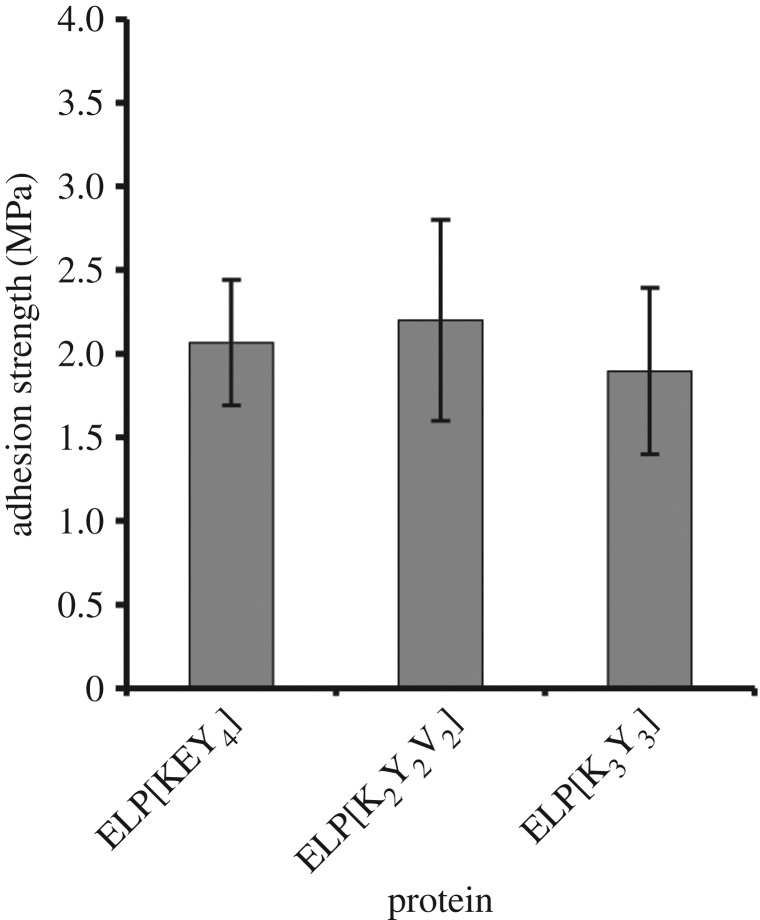
Effect of protein amino acid sequence on the bulk adhesion strength. Despite an approximately 10% variation in protein sequence, all of the ELPs possessed similar adhesion strengths when assessed by one-way ANOVA followed by Tukey's HSD post hoc analysis.

Three ELPs were used to elucidate the effect of amino acid composition on bulk adhesion strength: ELP[KEY_4_-48], ELP[K_3_Y_3_-48] and ELP[K_2_Y_2_V_2_-48]. The three ELPs had similar hydrophobicities based on the scale by Urry and co-workers [23,36], but had different numbers of charged residues (i.e. lysine and glutamic acid), which resulted in an overall sequence difference of approximately 10%. To control for the factor of size, all of these proteins had similar molecular weights of approximately 26 kDa. Bulk adhesion strengths of these proteins are shown in figure 7. All of the ELPs had statistically similar adhesion strengths.

**Figure 8.**
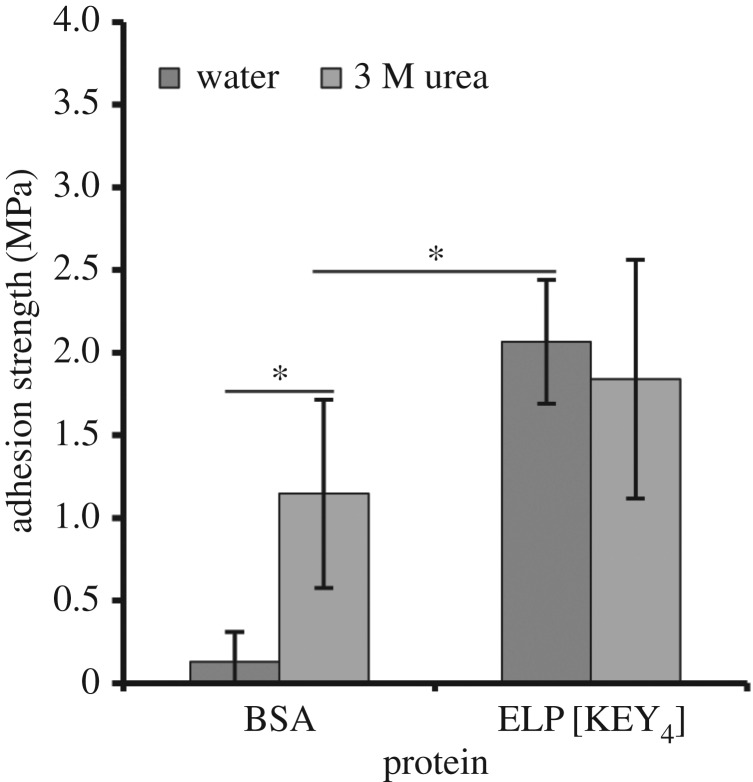
Effect of a denaturant (3 M urea) on bulk adhesion strength. The adhesion of the highly structured BSA protein was compared with the relatively unstructured ELP[KEY_4_-48] protein. The addition of the denaturant had a highly positive effect on BSA adhesion, whereas no effect was observed with ELP. Statistically significant differences (unpaired *t*-test, *p* *<* 0.05) are indicated by an asterisk.

Based on previous work with soy protein adhesives, the addition of a denaturant can increase adhesion strength due to disruption of protein structure [42]. Because ELPs are largely unstructured, however, the addition of a denaturant should not have a significant effect. Since denaturants have greater potential impact on a structured protein than on an unstructured one, the highly structured bovine serum albumin (BSA) protein was used here for a positive control. Thus, the effect of protein structure on bulk adhesion was examined by the addition of a denaturant (3 M urea) to ELP[KEY_4_-48] and BSA. Figure 8 shows that the presence of 3 M urea significantly increased the adhesion strength of BSA. However, even with this enhancement, BSA was still significantly weaker than ELP[KEY_4_-48] alone. Urea did not have an effect on the strength of ELP[KEY_4_-48]. Given that ELP[KEY_4_-48] was largely unstructured as assessed by CD, it was not surprising that urea did not have an effect on its adhesion strength.

**Figure 9.**
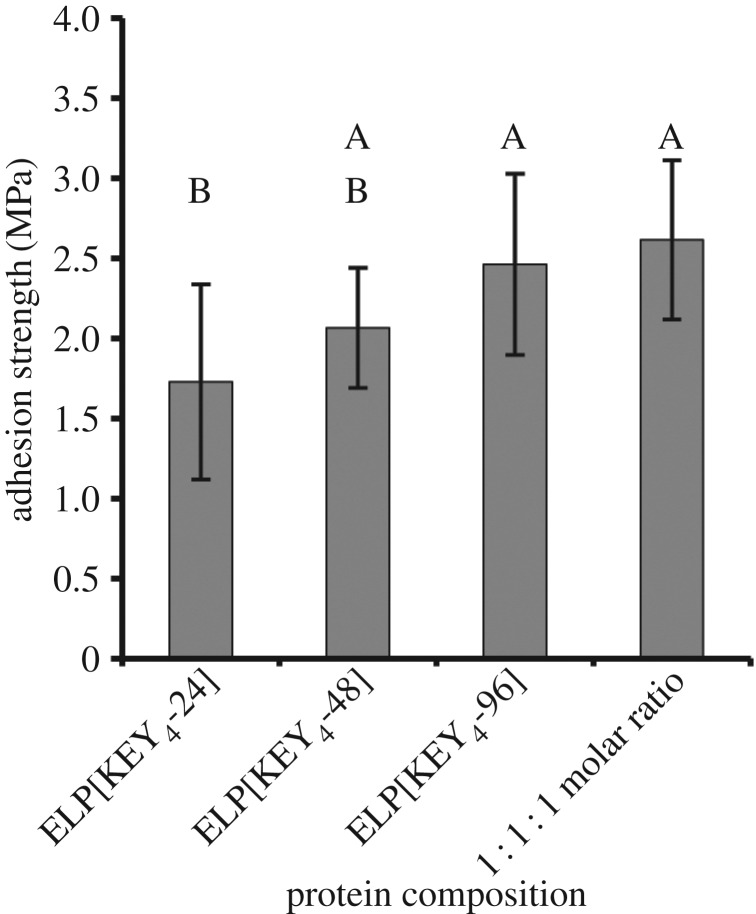
Effect of protein molecular weight on bulk adhesion strength. The bonding of ELP[KEY_4_-*n*] with 24, 48 and 96 pentapeptides was tested. To see the effect of a mixture of molecular weights, a 1 : 1 : 1 molar ratio of the three proteins was also tested. Generally, adhesion strength increased with molecular weight. The mixture of molecular weights resulted in a similar adhesion strength to that of either of the two longest proteins tested alone. Groups with identical letters are statistically similar (*p* *>* 0.05) when determined by one-way ANOVA followed by Tukey's HSD post hoc analysis.

The final factor investigated here was the effect of protein molecular weight, or length, on bulk adhesion strength (figure 9). The protein ELP[KEY_4_-*n*] was produced with 24, 48 and 96 repeats (15.5 kDa, 26.6 kDa and 48.8 kDa, respectively) to assess directly the effect of molecular weight. Adhesion strength of ELPs improved with increasing molecular weight. Furthermore, a 1 : 1 : 1 molar ratio (keeping the total mass constant) of the three protein sizes was also tested to determine the effect of a mixture of molecular weights because this strategy improved adhesion strength in a previous study with a mussel-mimetic polymer [32]. The mixture of molecular weights exhibited similar adhesion strength to the proteins with 48 and 96 pentapeptides.

### 3.4. Comparison with commercial adhesives

To put these results into a broader context, the adhesion strength of commercial protein-based adhesives was compared with that of the new proteins described here. Two commercial adhesives were chosen for comparison: Titebond Liquid Hide Wood Glue and Tisseel fibrin sealant. For each adhesive, total protein mass was kept consistent during testing (i.e. approx. 1.5 mg total protein per test). Results are shown in table 3. The adhesion strength of the hide glue was 2.0 ± 1.0 MPa, which was similar to the highest ELP strength measured in this study using the ELP[KEY_4_] 1 : 1 : 1 molar ratio (2.6 ± 0.8 MPa). The adhesion strength of Tisseel was significantly lower, however, at an average strength of 0.7 ± 0.3 MPa.

**Table 3.** Lap shear adhesion of recombinant elastomeric proteins compared with commercial adhesives.

adhesive	strength ± s.d. (MPa)^a^
hide glue	2.0 ± 1^A^
Tisseel	0.7 ± 0.3^B^
ELP[KEY_4_] 1 : 1 : 1 ratio^b^	2.6 ± 0.8^A^

^a^Groups with identical superscript capital letters are statistically similar (*p* *>* 0.05) when determined by one-way ANOVA followed by the Games–Howell post hoc analysis.

^b^ELP data are the same as in figure 9.

## 4. Discussion

Nature has created numerous protein-based glues for a wide variety of purposes. Interestingly, many of these protein adhesives have similar adhesion strengths and amino acid contents [14–17]. In this study, we systematically probed potential factors critical to protein-based adhesion strength to: (i) better understand the function of these adhesives in nature and (ii) aid in the design of future protein-based adhesives. We approached this problem by designing a system of recombinant ELPs because these proteins have similar amino acid compositions to natural protein glues and have been widely studied for structural biomaterials [14–17,19]. In addition, the use of recombinant proteins allows us to have precise control over sequence and molecular weight. Using these proteins, we examined the effect of various extrinsic and intrinsic factors on lap shear adhesion strength.

We first investigated the effect of extrinsic factors on the adhesion of a single protein, ELP[KEY_4_-48]. Our results suggested that neither pH nor concentration contributed to adhesion strength in any significant fashion, despite the fact that variations in pH affected protein surface charge, structure and solubility (see figure 2*a* and electronic supplementary material, figure S3), and variations in concentration affected solution viscosity and possibly bond thickness [40]. In prior literature, the contribution of pH to protein-based adhesion varies, whereas concentration is often a significant factor in strength.

Soy protein adhesion is quite sensitive to pH [43–45]. Processing pH affects protein solubility and the subsequent product. When applying the glue, a moderately high pH led to protein hydrolysis and increased adhesion strength [2]. An excessively high pH negatively affected the adhesive properties of soy, however [44,45].

The changes in protein properties resulting from pH can also affect the interactions between protein and substrate and thereby influence an adhesive bond. For example, the adsorption of a DOPA-less MAP varied greatly between pH 3 and 5.5 [46]. In general, adsorption of other proteins was highly dependent on pH [47]. On the other hand, adjusting the pH from 4 to 11 did not have an effect on the single-molecule adhesion of a silk-based protein [48].

Other adhesive systems suggest that protein concentration should affect adhesion strength. For a synthetic mussel-mimetic polymer, increasing the concentration from 75 to 1.2 g ml^−1^ led to a significant increase in adhesion strength [49]. The increase in strength was attributed to an increase in viscosity, which can affect bond thickness and resulting strength [40]. Viscosity and concentration are also central to soy protein adhesion. Strength increased up to an optimal concentration of approximately 100 mg ml^−1^ before decreasing at a concentration of approximately 140 mg ml^−1^ [50]. Viscosity also affected adhesion strength by altering the interaction between soy protein and the substrate [43,44,50].

The use of a cross-linker was also thought to be potentially important because bulk bonding depends on a balance between adhesive and cohesive forces [51]. A cross-linking agent can induce greater protein–protein interactions and thus, theoretically, reduce the possible protein–substrate interactions. In our recombinant protein system, however, varying the cross-linker ratio from 0× to 100× did not affect the adhesion strength of ELP[KEY_4_-48]. By contrast, the addition of a cross-linker had a strong effect on adhesion strength in systems that use DOPA [49,52–54]. Cross-linking agents have also been used to improve the adhesive properties of soy protein adhesives [2,44,55]. Notably, however, the adhesive proteins derived from the frog *N. bennetti* displayed no evidence of cross-linking during the curing process [14]. In our study, lysine residues could potentially contribute to adhesion via electrostatic attraction. Sacrificing the lysine residues through cross-linking with THP could have an opposing effect on adhesion, which could explain the lack of effect seen here. Future studies could examine the effect of other cross-linkers or the effect of cross-linkers in wet conditions.

The single most important extrinsic factor for our protein adhesives was moisture. When cured in a humid environment, the adhesion strength of ELP[KEY_4_-48] decreased by a factor greater than 4. When cure time and temperature were varied, adhesion strength increased with time and temperature until it reached a stable optimum around approximately 2 MPa. TGA suggested that the increase in strength was directly related to residual water content. These results were not unexpected; with few exceptions [17], bonding in wet environments is nearly always weaker than in a dry environment and remains one of the greatest challenges for adhesive engineering. For example, proteinaceous glue from *N. bennetti* also produced optimum strengths of approximately 1.7 MPa when completely dried [14]. It should be noted, however, that adhesion strength of our protein cured for long times (approx. 24 h) was not related to the cure temperature. This result was similar to that with a mussel-mimetic recombinant protein [53], but quite distinct from that with a mussel-mimetic polymer [49]. Soy protein adhesives demonstrate varying responses to cure temperature; in one study, increasing the cure temperature from 25°C to 100°C increased the strength from approximately 1.5 to approximately 2 MPa [50], whereas another study found that varying the temperature between 120°C and 160°C had no effect on strength [56].

Our recombinant protein system also allowed us to examine the effect of factors intrinsic to the proteins themselves, including amino acid composition, structure and molecular weight. To probe the effect of changes in protein sequence, we compared the bulk adhesion of several ELPs whose guest residues varied in their content of charged (lysine and glutamic acid) and hydrophobic (tyrosine and valine) residues while maintaining similar overall hydrophobicity. The ELP sequences differed by approximately 10% in the amino acid sequences and varied in isoelectric points from 6.38 to 10.23. However, there was little difference in adhesion strengths. A similar effect was seen with silk-ELPs [26]. In that study, cysteine, glutamic acid and tyrosine were each over-represented in different constructs, but there was no difference in protein surface adhesion when measured by a tape peel test. Likewise, other natural adhesive proteins demonstrated strong adhesion despite large variations in amino acid composition and overall hydrophobicity [14–17].

However, other types of protein adhesives have noted a strong influence of amino acid composition. For example, numerous studies of mussel-mimetic systems have demonstrated the potential importance of lysine residues in MAP adhesion [10–13]. In one study, increasing the cation content of a synthetic mussel-mimetic polymer from 0% to 7% increased the ambient adhesion strength from 2.4 to 2.8 MPa and doubled the underwater adhesion strength to a maximum value of 0.4 MPa [10]. Although hydrophilic lysine residues are thought to improve adhesion in MAPs, the adhesion strength of soy and sorghum adhesives has been correlated with increased hydrophobicity. It is thought that hydrophobic residues aid in repelling water from the adhesive bond [42,57].

Although amino acid composition varies among many natural adhesive proteins, many of these adhesives are unified by the over-representation of glycine, proline and/or serine [14–17]. Because glycine is over-represented in many of these natural adhesive proteins, many of them also lack significant secondary structure [14,15,58] and are thus similar to ELPs [39]. To determine the effect that protein structure might have on adhesion strength, we compared the adhesion strength of BSA and an ELP dissolved in water versus 3 M urea. The CD spectra (figure 2*a*) showed that ELP[KEY_4_-48] was largely unstructured (negative peak at 198 nm), but possessed slight secondary structure in the form of β-II turns (negative peak at 220 nm). By contrast, BSA was a highly structured globular protein. The addition of 3 M urea to BSA resulted in a significant improvement in adhesion strength, whereas it had no effect on the adhesion strength of ELP[KEY_4_-48]. The BSA results matched studies performed with soy adhesives. In general, denaturation of globular soy proteins is required to produce significant adhesion strength [2,43]. One of the most effective methods of soy protein denaturation is alkali treatment [45], although other chemical treatments have been used, including sodium dodecyl sulfate, sodium dodecylbenezene sulfonate, urea and guanidine hydrochloride [42,59,60]. In one study, the adhesion strength of soy protein increased with a denaturant concentration up to an optimum concentration of 3 M urea or 1 M guanidine hydrochloride. Even higher concentrations of either denaturant (8 M and 3 M, respectively) resulted in reduced adhesion strength [42]. Similar results were observed in another study: partial denaturation of soy protein with 1 M urea increased adhesion strength, whereas further denaturation with 3 M urea reduced adhesion strength [60]. Altogether, these results indicate that unstructured proteins may be beneficial to protein-based adhesion.

The final factor examined in this study was the effect of protein molecular weight. We produced one of our ELPs, ELP[KEY_4_-*n*], with *n* = 24, 48 or 96 elastin-like pentapeptides, which resulted in proteins of three different molecular weights: 15.5 kDa, 26.6 kDa and 48.8 kDa, respectively. The adhesion strength of these proteins increased with protein molecular weight. This result was similar to numerous other adhesive systems: poly(dimethylsiloxanes) (tested up to 68 000 g mol^−1^) [61,62], a mussel-mimetic polymer (tested up to 100 000 g mol^−1^) [32], silk-ELPs (tested up to 130 kDa) [26] and trypsinized soy protein isolate (mixtures of proteins with molecular weights ≤200 kDa) [4]. The bond strength enhancement is thought to be related to increased chain entanglement and elongation prior to breaking [32,62,63]. On the other hand, another study with soy protein adhesives showed increased adhesion strength after treatment with the protease trypsin, which should have reduced the average molecular weight of the soy proteins [64]. In addition, epoxidized natural rubber [65,66] and poly(vinyl alcohol) [67,68] demonstrated optimum adhesion at intermediate molecular weights (50 000 g mol^−1^ and 100 000 g mol^−1^, respectively), but this result may be due to increased surface wetting [62,66].

Because bonding derives from a balance between wettability and strength [65,66], optimum conditions could result from a blend of molecular weights [32]. In nature, protein adhesives from insects and amphibians are often a mixture of molecular weights [14–16]. In addition, mussels are known to adhere through a spatially organized combination of six adhesive proteins of varying molecular weights [69]. Proteins with lower molecular weights are found closer to the adhesive interface. When this idea was applied to a synthetic mussel-mimetic polymer, a blend of molecular weights possessed an adhesion strength similar to the average strength of the individual components [32]. Upon cross-linking, however, the mixture exhibited bonding in excess of the individual components. We investigated how a similar approach of mixing molecular weights would affect the adhesion strength of our protein adhesives. We mixed the three molecular weights of ELP[KEY_4_-*n*] in a 1 : 1 : 1 molar ratio. Unlike the mussel-mimetic polymer, however, we did not see a strength corresponding to the strength of the average molecular weight; instead, the blended protein mixture demonstrated equivalent strength to that of the two largest molecular weight proteins alone.

To bring greater context to our results, we also compared the adhesion strength of our proteins with those of two commercial protein adhesives: a wood glue derived from animal hide and Tisseel, a fibrin-based surgical sealant. Hide glue consists of long, non-reactive protein chains that interact non-specifically with the substrate [70], whereas fibrin sealants function by catalysing the formation of a fibrin clot via activation of fibrinogen by thrombin [71]. We hypothesized that ELPs would adhere by a similar mechanism to that of hide glue. The similar mechanisms may explain why, when tested using identical conditions with an equivalent mass of protein per test, our elastomeric proteins demonstrated equivalent adhesion strength to that of hide glue (table 3). Furthermore, our ELPs demonstrated superior adhesion strength to that of Tisseel. From this work, it can be said that unstructured ELPs were able to produce significant adhesion strengths that rivalled commercial glues and that these protein adhesives were not affected by variations in pH, concentration, cross-linking or sequence.

## 5. Conclusions

In this work, we examined the potential for elastomeric proteins to be used for adhesive materials. By using a system of ELPs, we were able to probe the critical factors related to adhesion strength. We found that, for a single protein, moisture content was a more significant factor than pH, concentration or cross-linking. In terms of protein design, protein length and structure had the most significant effect on adhesion strength. Finally, our proteins demonstrated significant adhesion strengths equivalent to or greater than two commercially available protein-based adhesives. These results have implications for the general understanding of natural and recombinant protein-based adhesion and for the design of future protein-based adhesives.

## Supplementary Material

Supplementary Material

## Data accessibility

Supporting data for this article have been uploaded as part of the electronic supplementary material.
