# Supplementary material for: Critical factors for the bulk adhesion of engineered elastomeric proteins
Source: R Soc Open Sci. 2018 May 9;5(5):171225. doi: 10.1098/rsos.171225 (PMC5990844; doi:10.1098/rsos.171225)
Supplement: Supplementary Material [file rsos171225supp1.docx]

**Supplementary Material**

**Critical factors for the bulk adhesion of engineered elastomeric proteins**

M. Jane Brennan,^1^ Sydney E. Hollingshead,^1^ Jonathan J. Wilker,^2,3^ and Julie C. Liu^1,4, *^

^1^Davidson School of Chemical Engineering, Purdue University, West Lafayette, IN 47907, USA

^2^Department of Chemistry, Purdue University, West Lafayette, IN 47907, USA

^3^School of Materials Engineering, Purdue University, West Lafayette, IN 47907, USA

^4^Weldon School of Biomedical Engineering, Purdue University, West Lafayette, IN 47907, USA

^*^ Corresponding author, Email: julieliu@purdue.edu

Supplementary Methods: Protein Expression

For each protein, the following bacterial expression strains were screened to identify the strain with highest expression: Rosetta2(DE3)pLysS (EMD Chemicals, Gibbstown, NJ), BL21(DE3)pLysS (courtesy of Dr. Chongli Yuan, Purdue University), BL21(DE3), and BL21-CodonPlus-(DE3)-RIPL (both courtesy of Dr. Jo Davisson, Purdue University). The strains chosen for expression of each protein are shown in Table 1. Cells were grown overnight at 37 °C in 2xYT medium containing appropriate antibiotics.

For ELP[KEY_4_-48] and ELP[K_3_Y_3_-48], overnight cultures were diluted at a 1:250 ratio into a fermentor (BioFlo 110, 14 L capacity, New Brunswick Scientific, Enfield, CT) with 10 L of Terrific Broth (TB) containing appropriate antibiotics. At an optical density (OD) of 4-6, protein expression was induced by 2.5 mM isopropyl *β*-d-1-thiogalactopyranoside (IPTG, EMD Chemicals). After culturing for an additional 1-3 h, cells were harvested by centrifugation at 8000*g* for 15 min at 4 °C.

For ELP[KEY_4_-24], ELP[KEY_4_-96], and ELP[K_2_Y_2_V_2_-48], overnight cultures were diluted at a 1:140 ratio into 4 L baffled flasks containing 1 L of 2xYT medium with appropriate antibiotics. Cells were cultured at 37 °C and 300 rpm. Once cell growth had reached an OD of *∼*1, protein expression was induced by 1 mM IPTG. Cells were then cultured for an additional 3 h and harvested by centrifugation at 3220*g* for 20 min at 4 °C.

Supplementary Methods: Protein Purification

ELP[KEY_4_-48] was purified by a temperature cycling method similar to those previously described [31]. The cell pellet was resuspended in 0.01 M sodium carbonate with *∼*0.1 mg each of deoxyribonuclease I, ribonuclease A, and phenylmethylsulfonylfluoride (PMSF). The pellet was then subjected to at least two freeze-thaw cycles and followed by sonication with a Misonix XL-2000 (Qsonica, Newtown, CT) for at least 90 cycles of 1 min sonication followed by 1 min cooling on ice. Next, a hot cycle was performed to induce the protein into a coacervate state. The pH of the cell lysate was adjusted to 8.6, heated at *∼*60 °C for 45 min, and centrifuged at 11000*g* for 45 min at 40 °C. The pellet from the hot cycle was then subjected to a cold cycle to resolubilize the target protein: the pellet was resuspended in 0.01 M sodium carbonate at 3 mL per gram of pellet, adjusted to pH *∼*11, cooled on ice, and then centrifuged at 11000*g* for 45 min at 4 °C. Beginning with the supernatant from this cold step, an additional hot cycle and cold cycle were performed, but the second heated pellet was resuspended at 20 mL per gram of pellet.

The ELP[K_3_Y_3_-48] was purified similarly to ELP[KEY_4_-48] but with both hot and cold cycles at pH 7.4 and the addition of sodium chloride to a final concentration of 1 M during the hot cycles.

All of ELP[KEY_4_-24], ELP[KEY_4_-96], and ELP[K_2_Y_2_V_2_-48] were purified using denaturing nickel affinity chromatography. Cell pellets were resuspended in Buffer B (8 M urea, 100 mM NaH_2_PO_4_, 100 mM Tris-Cl, pH 8.0), subjected to at least two freeze-thaw cycles, and sonicated as above. The cell lysate was centrifuged at 10000*g* for 45 min at 4 °C to remove cell debris. The supernatant was mixed with nickel-nitrilotriacetic acid (Ni-NTA) agarose (QIAGEN, Valencia, CA) at a concentration of 2 mL of lysate per mL of Ni-NTA and loaded onto a chromatography column (Flex-Column, Kimble Chase, Vineland, NJ). This column was incubated at 37 °C and 100 rpm for 1 h to allow the desired protein to bind to the Ni-NTA. Next, undesired proteins were allowed to drip out of the column by gravity flow, and the column was washed with 3 bed volumes of Buffer C (8 M urea, 100 mM NaH_2_PO_4_, 100 mM Tris-Cl, 10 mM imidazole, pH 6.3). Purified protein was eluted with 5 bed volumes of Buffer D/E (8 M urea, 100 mM NaH_2_PO_4_, 100 mM Tris-Cl, pH 5.5).

Purified proteins were dialyzed extensively against 5% acetic acid at 4 °C to remove

salts and were then lyophilized. Protein expression and purification were confirmed by analysis with sodium dodecyl sulfate-polyacrylamide gel electrophoresis (SDS-PAGE) and Western blot using standard techniques [30]. Proteins were detected using an anti-T7 tag antibody conjugated to horseradish peroxidase (EMD Chemicals, Gibbstown, NJ) with a colorimetric substrate (3,3’,5,5’-tetramethylbenzidine, Kirkegaard & Perry Laboratories, Gaithersburg, MD). SDS-PAGE gels were stained with Coomassie Brilliant Blue R-250. Protein purity was assessed through densitometry analysis of SDS-PAGE gel images with ImageJ software (NIH, Bethesda, MD). Purified protein molecular weights were verified by matrix-assisted laser desorption/ionization-time of flight (MALDI-TOF) mass spectrometry (Dr. Connie Bonham, Campus-Wide Mass Spectrometry Center, Purdue University). Briefly, the MALDI mass spectra were obtained on a Voyager DE-Pro TOF mass spectrometer (Applied Biosystems, Framingham, MA) in the linear mode with delayed extraction. The matrix was sinapinic acid. Positive-ion spectra were obtained with an acceleration voltage of 25000 V. Additionally, purified protein amino acid compositions were verified by the Molecular Structure Facility at the University of California, Davis.


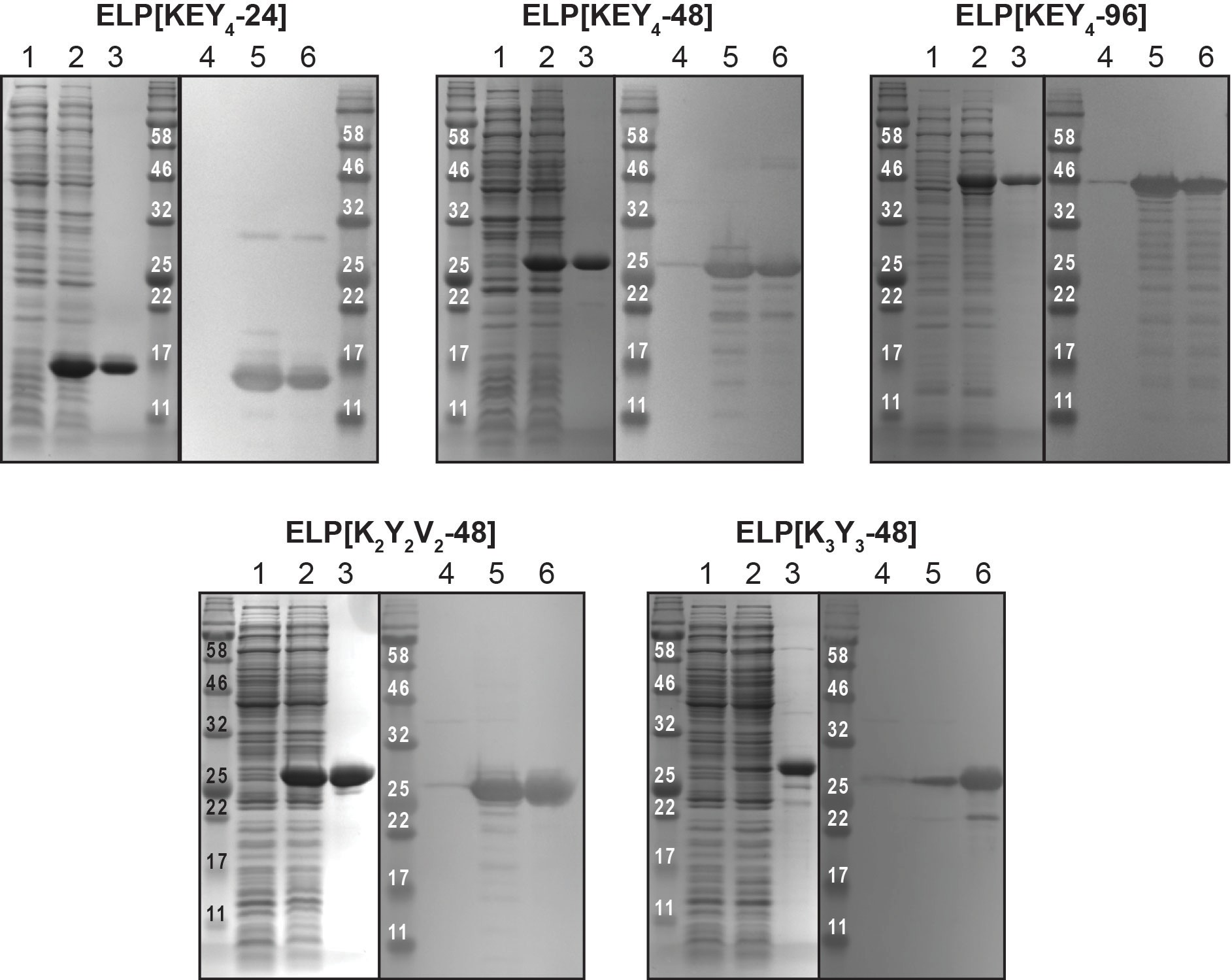


**Figure S1**. SDS-PAGE gels and Western blots showing expression and purified protein samples for each protein in this study. Lanes 1-3 correspond to SDS-PAGE gels, whereas lanes 4-6 correspond to Western blots. Lanes 1 and 4 show culture samples prior to induction of expression with IPTG. Lanes 2 and 5 show culture samples at harvest with over-expressed proteins. Lanes 3 and 6 show purified protein at *∼*1 mg/mL. Protein standard bands are labeled with masses in kDa. The calculated molecular weights of the proteins are shown in Table 1.


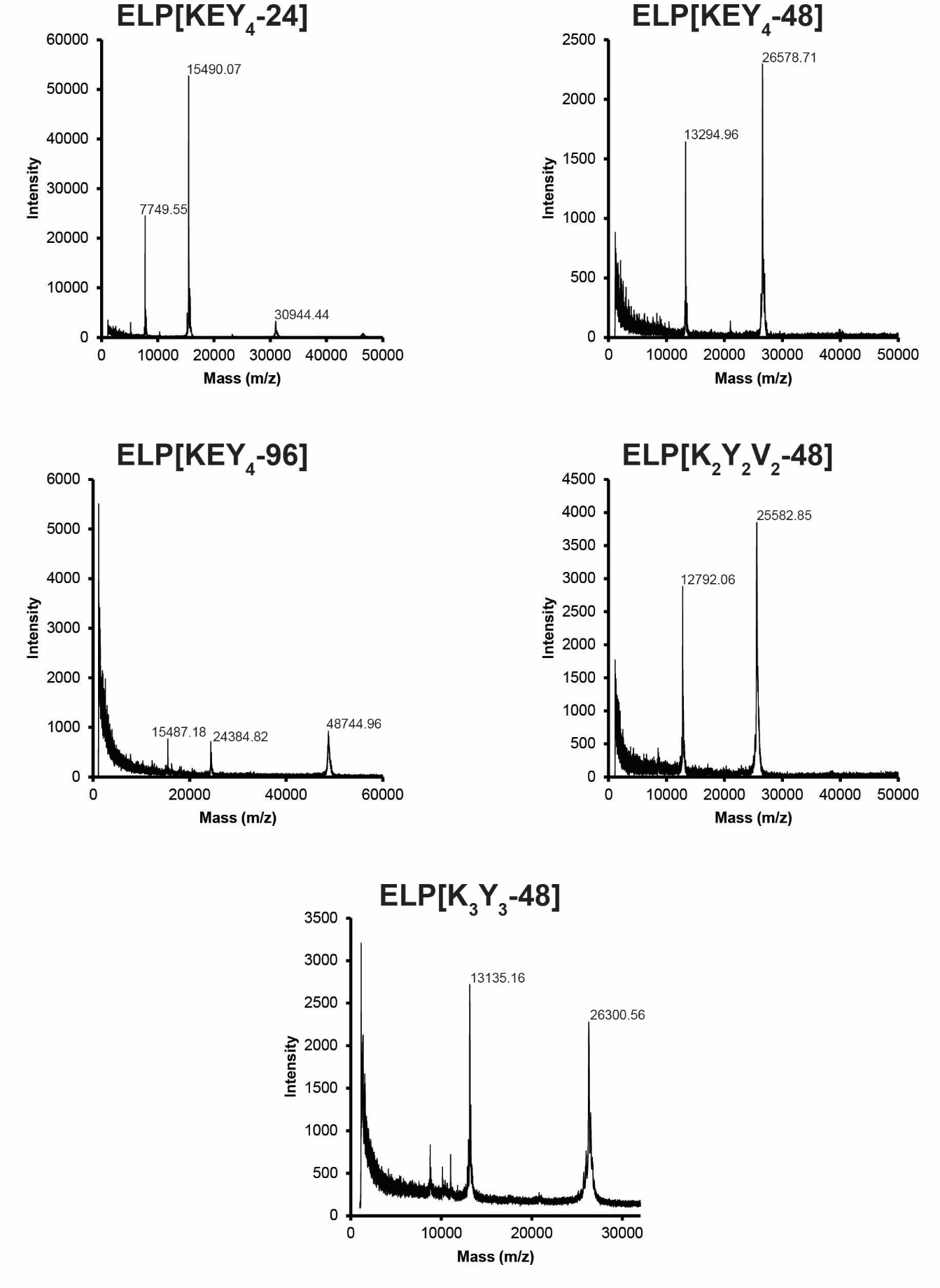


**Figure S2**. MALDI-TOF spectra of the proteins used in this study. Spectra for all proteins exhibited peaks within 0.13% of the expected protein molecular weights. Many spectra also contained multiply charged ion peaks, such as the double charged ion peak that appears at *∼*50% of the protein expected molecular weight. In addition, the spectrum for ELP[KEY_4_-24] exhibits a peak at twice the expected molecular weight corresponding to a protein dimer.

**Table S1**. Amino acid analysis of ELP[KEY_4_-24].

| **Amino Acid** | **Observed mol%** | **Expected mol%** |
| --- | --- | --- |
| ASX | 4.00 | 3.90 |
| THR | 1.33 | 1.30 |
| SER | 0.65 | 0.65 |
| GLX | 4.42 | 4.55 |
| PRO | 15.83 | 16.23 |
| GLY | 34.57 | 34.42 |
| ALA | 1.40 | 1.30 |
| VAL | 15.72 | 16.23 |
| ILE | 0.04 | 0.00 |
| LEU | 2.01 | 1.95 |
| TYR | 10.40 | 10.39 |
| PHE | 0.00 | 0.00 |
| HIS | 4.56 | 4.55 |
| LYS | 3.59 | 3.25 |
| ARG | 1.47 | 1.30 |

**Table S2**. Amino acid analysis of ELP[KEY_4_-48].

| **Amino Acid** | **Observed mol%** | **Expected mol%** |
| --- | --- | --- |
| ASX | 3.41 | 2.17 |
| THR | 1.42 | 0.73 |
| SER | 1.64 | 0.36 |
| GLX | 5.49 | 4.01 |
| PRO | 16.27 | 17.88 |
| GLY | 32.91 | 36.86 |
| ALA | 2.02 | 0.73 |
| VAL | 15.26 | 17.88 |
| ILE | 0.44 | 0.00 |
| LEU | 2.14 | 1.09 |
| TYR | 10.20 | 11.68 |
| PHE | 0.53 | 0.00 |
| HIS | 2.68 | 2.55 |
| LYS | 3.83 | 3.28 |
| ARG | 1.67 | 0.73 |

**Table S3**. Amino acid analysis of ELP[KEY_4_-96].

| **Amino Acid** | **Observed mol%** | **Expected mol%** |
| --- | --- | --- |
| ASX | 1.77 | 1.17 |
| THR | 0.75 | 0.39 |
| SER | 0.46 | 0.19 |
| GLX | 3.90 | 3.70 |
| PRO | 17.93 | 18.87 |
| GLY | 37.44 | 38.33 |
| ALA | 0.89 | 0.39 |
| VAL | 17.70 | 18.87 |
| ILE | 0.19 | 0.00 |
| LEU | 0.99 | 0.58 |
| TYR | 11.68 | 12.45 |
| PHE | 0.06 | 0.00 |
| HIS | 1.72 | 1.36 |
| LYS | 3.72 | 3.31 |
| ARG | 0.82 | 0.39 |

**Table S4**. Amino acid analysis of ELP[K_2_Y_2_V_2_-48].

| **Amino Acid** | **Observed mol%** | **Expected mol%** |
| --- | --- | --- |
| ASX | 2.45 | 2.19 |
| THR | 0.84 | 0.73 |
| SER | 0.41 | 0.36 |
| GLX | 1.15 | 1.09 |
| PRO | 17.53 | 17.88 |
| GLY | 37.13 | 36.86 |
| ALA | 0.84 | 0.73 |
| VAL | 22.74 | 23.72 |
| ILE | 0.05 | 0.00 |
| LEU | 1.17 | 1.09 |
| TYR | 5.72 | 5.84 |
| PHE | 0.00 | 0.00 |
| HIS | 2.70 | 2.55 |
| LYS | 6.45 | 6.20 |
| ARG | 0.80 | 0.73 |

**Table S5**. Amino acid analysis of ELP[K_3_Y_3_-48].

| **Amino Acid** | **Observed mol%** | **Expected mol%** |
| --- | --- | --- |
| ASX | 2.48 | 2.19 |
| THR | 0.93 | 0.73 |
| SER | 0.48 | 0.36 |
| GLX | 1.44 | 1.09 |
| PRO | 17.20 | 17.88 |
| GLY | 36.48 | 36.86 |
| ALA | 1.13 | 0.73 |
| VAL | 17.18 | 17.88 |
| ILE | 0.24 | 0.00 |
| LEU | 1.26 | 1.09 |
| TYR | 8.39 | 8.76 |
| PHE | 0.17 | 0.00 |
| HIS | 2.17 | 2.55 |
| LYS | 9.65 | 9.12 |
| ARG | 0.80 | 0.73 |


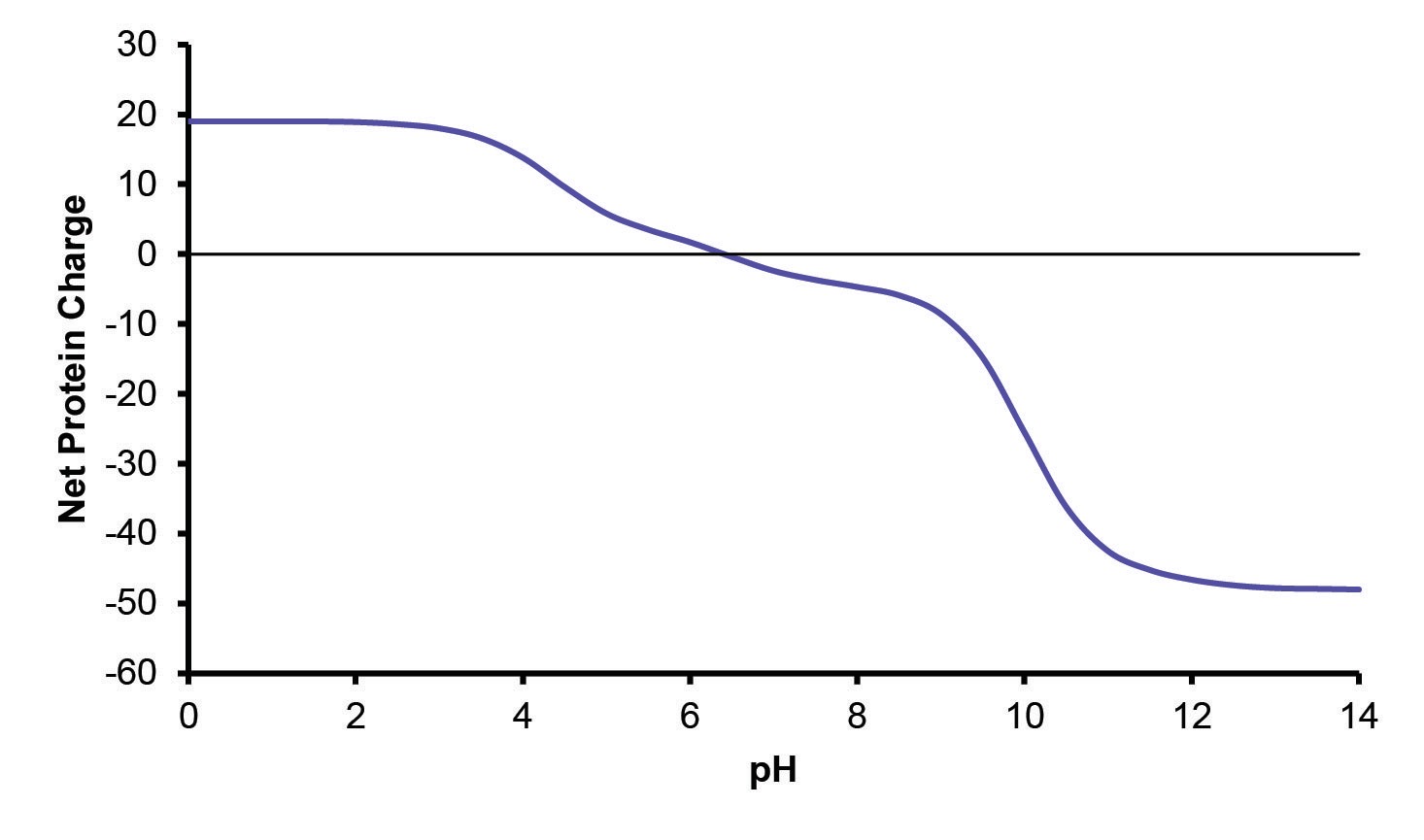


**Figure S3**. Estimated net charge vs. pH for ELP[KEY_4_-48]. An online calculator (available at http://protcalc.sourceforge.net/) was used to estimate charge based on the pKa values of the amino acids.


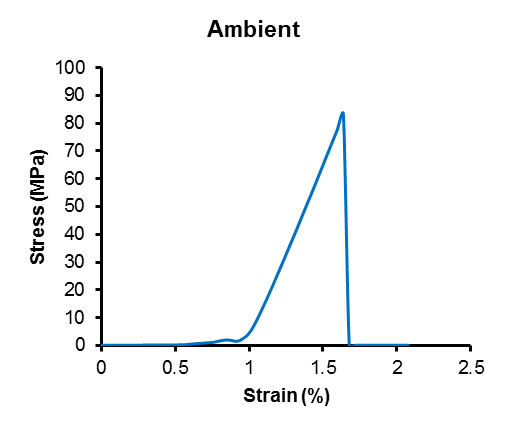

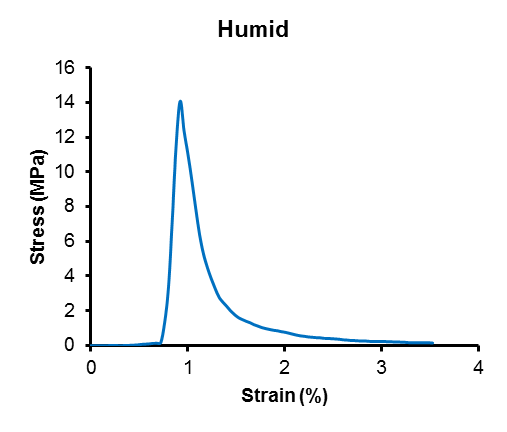


**Figure S4.** Representative stress-strain curves for ELP[KEY_4_-48] under ambient (left) and humid (right) conditions (150 mg/mL, pH 3, 6 h cure). Curves from same data as in Figure 5.

**Table S6.** Representative mechanical properties of ELP[KEY_4_-48] under ambient and humid conditions (150 mg/mL, pH 3, 6 h cure) calculated from same data as in Figure 5.

| **Condition** | **Young’s Modulus (MPa)** | **Toughness (MJ/m^3^)** |
| --- | --- | --- |
| Ambient | 113.4 ± 15.9 | 22.5 ± 5.6 |
| Humid | 60.9 ± 17.8 | 5.0 ± 1.2 |
